# Comparative Evaluation of Cocoa Bean Husk, Ginger and Chlorhexidine Mouth Washes in the Reduction of Steptococcus Mutans and Lactobacillus Count in Saliva: A Randomized Controlled Trial

**DOI:** 10.7759/cureus.4968

**Published:** 2019-06-21

**Authors:** Shrimathi S, Umesh Kemparaj, Sangeeta Umesh, Muthu Karuppaiah, Palanivel Pandian, Krishnaveni A

**Affiliations:** 1 Public Health Dentistry, Best Dental Science College and Hospital, Madurai, IND; 2 Pharmacognosy, College of Pharmacy, Madurai Medical College, Madurai, IND

**Keywords:** ginger, cocoa bean husk, chlorhexidine, s.mutans, lactobacillus, mouth rinse

## Abstract

Introduction

Dental caries is a ubiquitous bacterial infection that has afflicted people for years. *Streptococci mutans *(*S. mutans*) are markers of dental caries and the population of *S. mutans* in the saliva is directly related to the number of surfaces colonized by them. Any intervention that can inhibit their growth and survival will negatively impact the initiation and progress of caries. Various antimicrobial agents have been tested against these microorganisms. The goal of this study was to assess the efficacy of cocoa bean husk, ginger, and chlorhexidine mouth rinse on *S. mutans *and Lactobacillus.

Materials and methods

We conducted a randomized controlled trial involving patients aged 18 to 25 years from July to September 2018. The study population was allocated into three groups. Each group received either cocoa bean, ginger, or chlorhexidine mouth rinses. The study followed a Latin square design. Study participants were instructed to use the assigned mouth rinse once daily for seven days. We collected saliva samples to measure* S. mutans* and Lactobacillus populations.

Results

Cocoa bean husk and chlorhexidine rinses produced a significant reduction of* S. mutans* (p < 0.05). The ginger-based rinse significantly reduced the Lactobacillus population (p < 0.05).

Conclusion

Our findings indicate these natural mouth rinses offer promising anticariogenic and antiplaque efficacy as cost-effective alternatives to traditional mouth rinses.

## Introduction

Oral health is a reflection of one’s general health. Among oral diseases, dental caries is the most common microbial infection and remains a clinical challenge. The main cause of dental caries is attributed to dental plaque [1]. The oral flora consists of 350 cultivable species [2]. The imbalance between preventive measures and increased prevalence of the disease has resulted in an enormous burden on society.

Dental caries is an irreversible disease which affects the tooth structure. Reducing Streptococci load in the oral cavity is a practical step to lower the incidence of dental caries [3]. Many plaque control agents possess promising antimicrobial action.

Mouthwashes used in dentistry for preventative and therapeutic purposes act by chemomechanical action; Chlorhexidine is the most potent chemotherapeutic agent and is the gold standard in reducing *S. mutans* and plaque. Adverse effects are associated with chlorhexidine, which led to a focus on potential natural alternatives with high antibacterial effects but less toxicity than chlorohexidine [2]. Some potential herbal products were used in India for the treatment of various ailments, and recent commercial use of these products in toothpaste and oral irrigation delivery has increased [4]. Examples of potential natural alternatives to chlorohexidine are cocoa bean and ginger. Cocoa bean (*Theobroma cacao*), the raw ingredient of chocolate, belongs to the Malvaceae family has an antibacterial activity [5]. The main substance of polyphenols found in cocoa bean husk is catechin and epicatechin. In addition to these, there are free fatty acids such as oleic acid and linoleic acid that showed strong bactericidal activity against mutans streptococci [6]. Ginger (*Zingiber officinale*) is native to south-eastern Asia and belongs to the Zingiberaceae family [7]. While ginger is used primarily as a condiment, it seems to be effective against many oral diseases. The main active ingredients of ginger are phenolic compounds (i.e., gingerol and shogaol), sesquiterpene hydrocarbons, and oleoresins [8].

We conducted this study to assess safe and cost-effective mouthwashes for the prevention of oral diseases. Few studies have been conducted that compare the antimicrobial efficacy of chlorhexidine, cocoa bean husk, and ginger. Therefore, our goal was to compare the antimicrobial effects of Chlorhexidine, cocoa bean husk, and ginger mouth rinse on *S. mutans* and Lactobacillus levels in the saliva.

## Materials and methods

We conducted a double-blinded, randomized controlled crossover study (Latin square design) to compare the effectiveness of ginger, cocoa bean husk, and chlorhexidine mouth rinse on* S. mutans* and Lactobacillus levels in saliva. Ethical approval was obtained from the Institutional review board of Best Dental Science College, Madurai. Study participants were selected from the student body of the Dental College.

Based on previous study data the mean difference of *S. mutans* reduction in the study and control groups was anticipated as 0.45 [5]. Fixing the confidence interval and power of the study to 95% the sample size was calculated as 23 participants per group. Considering possible attrition of approximate 10%, two subjects were added in each group. The final sample was calculated as 25 subjects per group. The participants were students aged 18 to 25 years who were healthy and provided consent for inclusion. We excluded students who had received antibiotic therapy three weeks before the start of the study, had used a topical fluoride application, or mouthwash 48 hours prior to the study. Also excluded were any students with systemic illness, mental illness, or physical limitations and those with a history of allergy to any of the mouth rinse components used in the study. 

Students who met the inclusion criteria were randomized into one of three groups through lottery method, receiving either a 12.5% ginger rinse (Group A, GING), a 0.2% chlorhexidine rinse (Group B, CHX), or a 0.5% cocoa bean husk rinse (Group C, COCOA) respectively.

Preparation of the mouth rinses

The cocoa bean husk (The Campco, Ltd., Karnataka) and ginger (from a local market) products were washed, dried for one week, and ground to a fine powder.

The extract was obtained using a Soxhlet apparatus. We used 10 g of powder (either ginger or cocoa bean husk) and 100 ml of 70% ethanol a solvent. We obtained the extracts by evaporating the solvent via a rotary evaporator maintained at 80ºC for eight hours. We diluted 50 ml of cocoa bean husk extract and 125 ml of ginger extract in 100 ml of sterile distilled water. We added 0.1% peppermint oil added as a flavoring and saccharine as sweetening agent to obtain the desired concentration. The mouth rinses were prepared by the Pharmacognosy Department at the Government Pharmacy College in Madurai.

Distribution of the mouth rinse

**Figure 1:**
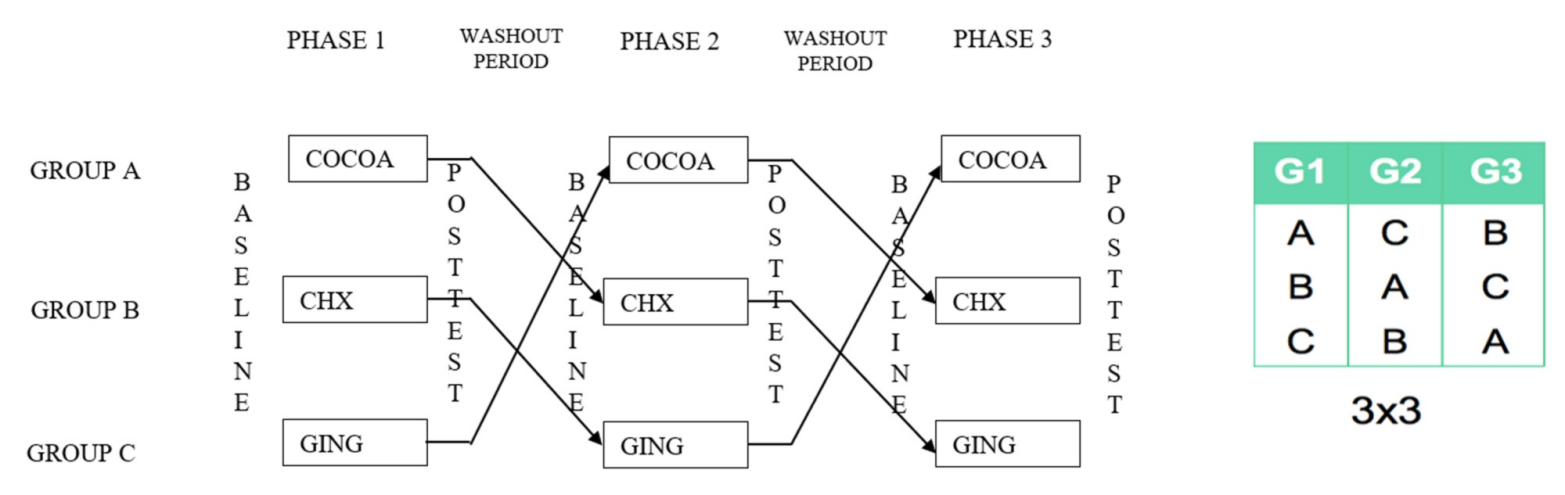
Latin square design depicting the sequelae of phases

The study was conducted in three phases and monitored by the principal investigator. The three groups received different mouth rinses packed in 100 ml opaque bottles marked with unique codes for data masking/study blinding. Participants were instructed to use the mouth rinse (10 ml) once per day for seven days in different phases with a washout period of 15 days, according to the Latin square design (Figure 1).

Before each phase, participants’ unstimulated saliva samples were collected in sterile containers and transferred to the laboratory for microbiological analysis. The collected samples were also masked to group information and stored in a cold storage box and analyzed within one hour of collection. Microbiological analysis was performed at the Department of Microbiology in the American College in Madurai. At the end of each phase, we determined the mean colony forming units (CFUs).

Statistical analysis

We used IBM SPSS Statistics for Windows, Version 22.0 (IBM Corp., Armonk, NY) to analyze the data. We performed an analysis of variance followed by post hoc analysis. We used paired and unpaired t-tests to determine significance. P-values of <0.05 were considered statistically significant.

## Results

A total of 75 students were included in the study (32 men and 43 women). We noted statistically significant reductions of *S. mutans* populations in all the three phases. The group of students using chlorhexidine solution had a greater reduction in *S. mutans* counts throughout the study than students using cocoa bean and ginger rinses (p < 0.05; Table 1). The mean scores of colony-forming units (CFUs) recorded at the end of each phase of the intervention was consistently lower in cocoa bean husk, ginger, and chlorhexidine group.

**Table 1:** Inter- and intra-group comparison of Streptococcus mutans values between the three groups at all three phases of intervention Colony-forming units per microlitre (CFUs/uL).

Group	Variables	1^st^ Phase			2^nd^ Phase			3^rd^ Phase		
		Mean (CFUs/uL)	SD	Sig. (two-tailed)	Mean (CFUs/uL)	SD	Sig. (two-tailed)	Mean (CFUs/uL)	SD	Sig. (two-tailed)
Cocoa Bean Husk	Baseline	2.108	.952	.032	1.644	1.652	.021	2.444	1.781	.023
	Final	1.610	.792		.909	.730		1.812	1.477	
Chlorhexidine	Baseline	2.473	.812	.001	2.004	1.074	.014	1.672	.683	.013
	Final	1.904	.547		1.372	.771		1.138	.757	
Ginger	Baseline	2.676	.980	.040	1.161	.845	.044	1.589	1.002	.048
	Final	2.108	.692		.832	.734		1.274	.887	
Inter group p-value		0.041			0.004			0.043		

Lactobacillus count was significantly reduced in the chlorhexidine and ginger groups but was only slightly reduced in the cocoa bean husk-derived rinse group (Table 2).

**Table 2:** Inter- and intra-group comparison of Lactobacillus values between the three groups at all three phase of intervention Colony-forming units per microlitre (CFUs/uL).

Group	Variables	1^st^ Phase			2^nd^ Phase			3^rd^ Phase		
		Mean (CFUs/uL)	SD	Sig. (2-tailed)	Mean (CFUs/uL)	SD	Sig. (2-tailed)	Mean (CFUs/uL)	SD	Sig (2-tailed)
Cocoa bean husk	Baseline	.210	.671	.247	.292	.699	.953	.3308	.77371	.922
	Final	.116	.300		.286	.573		.3132	.48642	
Chlorhexidine	Baseline	.693	1.469	.037	.269	.572	.048	.6360	.88292	.012
	Final	.0484	.102		.081	.282		.1168	.30037	
Ginger	Baseline	.636	.882	.021	.454	.677	.003	.4544	.67794	.003
	Final	.313	.486		.001	.003		.0016	.00374	
Inter group p value		0.019			0.02			0.005		

The inter-group comparison shows a minor difference in mean *S. mutans* levels between the three groups (p=0.54). We noted a higher reduction in the cocoa bean husk group (mean, 0.60 ± 1.25) followed by chlorhexidine (mean, 0.52 ± 1.04), and ginger (mean, 0.40 ± 0.97; Table 3).

**Table 3:** Inter-group comparison of S. mutans in all three phases of intervention Colony-forming units per microlitre (CFU-s/uL).

Group	Paired Differences				Sig. (two-tailed)
	Mean (CFUs/uL)	SD	95% CI		
			Lower	Upper	
Chlorhexidine	.5248	1.04010	.2855	.7641	.540
Cocoa husk	.6009	1.25053	.2855	.7641	
Ginger	.4040	.97460	.1798	.6282	

The inter-group comparison shows a significant mean difference in Lactobacillus levels (p=0.003). The higher reduction was observed in the chlorhexidine group (mean, 0.45 ± 1.04) followed by the ginger group (mean, 0.40 ± 0.66), and cocoa husk group (mean, 0.039 ± 0.64; Table 4).

**Table 4:** Inter-group comparison of Lactobacillus in all three phases of intervention Colony-forming units per microlitre (CFUs/uL).

Group	Paired Differences				Sig. (2-tailed)
	Mean (CFUs/uL)	SD	95% CI		
			Lower	Upper	
Chlorhexidine	.4509	1.04183	.2112	.6906	.003
Cocoa husk	.0395	.64267	-.1084	.1873	
Ginger	.4095	.66478	.2565	.5624	

## Discussion

Oral health is an integral part of a person’s overall health and quality of life. The paramount causative factor for oral diseases is dental plaque. The formation of plaque on the tooth surface occurs via the synthesis of glucan polymers and glucan binding proteins catalyzed by glucosyltransferases [9]. The progression of initial adherence of bacteria and subsequent accumulation by growth and inter-bacterial adherence lead to the coating of the tooth surface with a complex micro-community leading to the destruction of hard enamel tissue [10].

Herbal oral hygiene products may offer similar benefits to traditional oral hygiene products but with the potential for reduced adverse effects [11]. Several plant extracts exhibit promising antimicrobial effects both in in vitro and in vivo [12]. Cocoa bean husk mouth rinse possesses anti-glucosyltransferase and antibacterial activity which is effective in reducing *S. mutans* and plaque. The glucosyltransferases present in cocoa husk extract inhibits the adhesion of the *S. mutans* to saliva-coated hydroxyapatite and reduces the formation of dental plaque [5]. The active ingredients of ginger (gingerol and shogaol) possess antibacterial activity against *S. mutans* and Lactobacillus that could reduce dental caries through the inhibition of glucan synthesis and adherence [9, 13].

In our study, a low-concentration chlorhexidine mouth rinse (0.2%) caused a significant reduction of *S. mutans* population, which aligns with reports by Sari et al. [14] and Babu et al. [15]. However, our finding of a significant reduction of Lactobacillus contrasted with the Sari et al. study, which reported no difference in Lactobacillus levels [14].

Our cocoa bean husk mouth rinse results (significant reduction of *Streptococcus mutans*) align with the results reported by Srikanth et al. [5], Babu et al. [15], and Mustamin et al. [16], who used 0.1% cocoa bean husk mouth rinse twice daily in their study design, and we used 0.5% cocoa bean husk mouth rinse once daily, suggesting that increasing the concentration and decreasing the frequency of rinses can yield similar results. Reducing the frequency of daily mouth rinsing make the patients more comfortable and increases their compliance. The antimicrobial effects of ginger seen in our study align with findings reported by Al-Duboni et al. who found that ginger mouth rinse yielded a greater reduction in lactobacilli than *S. mutans* [17].

Herbal medications have been introduced as an alternative medicine to prevent and treat oral diseases. Widespread use of such products among the population will be beneficial even to the low socioeconomic status patients. These products were cost-effective and have shown to pose minimal side effects [18].

This study is the first of its kind to compare the effect of cocoa bean husk, ginger, and chlorhexidine on *S. mutans* and Lactobacillus. However, our study had several limitations. Our sample size was small, and the washout period was short. The taste and color of the rinses inhibited full masking of the study design. Further studies are needed to evaluate long-term effects and efficacy in a wider range of age groups.

## Conclusions

Mouth rinses derived from ginger and cocoa bean husks were effective in reducing CFUs/uL of *S. mutans* to a degree similar to the current gold standard for oral rinses, chlorhexidine. A rinse derived from ginger was equally efficacious in reducing the CFUs/uL of Lactobacillus. Ginger is a promising anticariogenic and antimicrobial mouth rinse active ingredient and may offer a lower-cost yet safe caries inhibitory agent compared to traditional mouth rinses.
